# Transscleral tunnel incision related arterial hemorrhage in 23-gauge Vitrectomy: case report

**DOI:** 10.1186/s12886-018-0677-6

**Published:** 2018-01-17

**Authors:** Bingqian Liu, Yonghao Li, Tao Li, Ying Lin, Wei Ma, Lin Lu

**Affiliations:** State Key Laboratory of Ophthalmology, Zhongshan Ophthalmic Center, Sun Yat-sen University, Guangzhou, Guangdong 510060 China

**Keywords:** Small gauge vitrectomy, Tunnel incision related complication, arterial bleeding, Ciliary artery, Case report

## Abstract

**Background:**

Transscleral tunnel incisions are commonly made to avoid postoperative leakage in small gauge sutureless vitrectomy. We present an unreported intraoperative complication, tunnel incision related arterial hemorrhage from sclerotomy, in 23-gauge (23G) vitrectomy.

**Case presentation:**

Two cases of intraocular arterial hemorrhage from superonasal sclerotomy were observed at the beginning of vitrectomy. The bleeding filled the vitreous cavity quickly and gushed out from the incision port after the involved supronasal cannula was removed. The active bleeding seemed not to stop spontaneously. We controlled the active bleeding by relocating the involved cannula, elevating the intraocular pressure and compressing the sclera wound. Post-operative intraocular hemorrhage from the sclerotomy was not found in any of the two cases.

**Conclusions:**

We suggest that the bleeding was from injured ciliary artery when the incision crossed 3 or 9 o’clock accidently. Surgeons might avoid this complication by locating the superior incisions away from the horizontal axis, and should be aware the proper management.

**Electronic supplementary material:**

The online version of this article (dio: 10.1186/s12886-018-0677-6) contains supplementary material, which is available to authorized users.

## Background

Sutureless is an important aspect of small gauge vitrectomy [1–3]. Oblique or biplanar incisions are recommended by many surgeons in order to create a watertight sclera tunnel, thus reducing the risk of postoperative hypotony associated complications [1–6].

## Case presentation

**Fig. 1:**
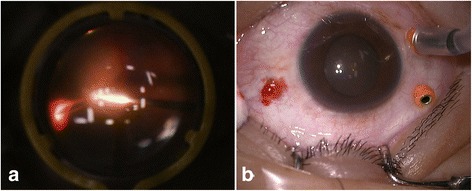
Transscleral tunnel incision related arterial hemorrhage. **a** Arterial hemorrhage from the superonasal incision observed through a wide angle viewing lens. **b** Blood gushed out through the transscleral incision after the cannula was removed

We made tunnel sclerotomies with trocar blades (Alcon Laboratories, Inc., TX, USA) using a biplanar technique for 23G vitrectomy. We present two cases of intraocular arterial hemorrhage through superonasal incision port at the beginning of vitrectomy (Fig. 1a). Neither of the two patients showed blood coagulation dysfunction in preoperative blood test.

### Case 1

A 52 year old Chinese male patient had a macular hole, confirmed by optical coherence tomography, in his left eye, lasting for 6 months. No systemic disease was recorded. His preoperative best corrected visual acuity (BCVA) was 0.1. Three port 23G vitrectomy was performed. We tried to insert the superior cannulars at about 2 and 10 o’clock, but actually the superonasal cannular seemed to be placed from 10 to 9 o’clock, crossing the horizontal axis. There was no prominent hemorrhage in the conjunctiva or episclera at incision sites after placing the trocars. A metal ring was then sutured at the limbus followed by placing a glass lens. At the beginning of vitrectomy, small volume of hemorrhage was observed, and soon the bleeding was found active and filled the vitreous cavity. Elevating the perfusion pressure could not stop the increase of vitreous hemorrhage. Considering that the bleeding was from ciliary artery, we relocated the superonasal cannula and observed blood gushed out from the initial incision wound. Compressing the sclera wound site and elevating the intraocular pressure for about 10 min stopped the bleeding finally. The vitreous hemorrhage was cleared. The internal limiting membrane was peeled and C3F8 (12%) tamponade was performed (Additional file 1). No further intraocular hemorrhage was observed postoperatively. Post-operative BCVA was 0.25 at three-months.

### Case 2

A 43-year-old male Chinese patient had proliferative diabetic retinopathy, diagnosed by history of diabetic disease and fundus imaging, in his right eye, lasting for 2 months. His visual acuity was finger counting at 1 m. Intravitreous injection of anti-vascular endothelial growth factor agent (ranibizumab, 0.5 mg) was performed 1 week prior to 23G vitrectomy. At the beginning of vitrectomy, active hemorrhage was observed from the superonasal incision site through non-contacting viewing lens (Fig. 1a). We removed the superonasal trocar (possibly crossing the horizontal axis) and observed blood gushed out from the incision wound (Fig. 1b). Compressing the sclera wound site and elevating the intraocular pressure for about 5 min stopped the bleeding. The cannula was reinserted with a trocar at adjacent site, and the vitreous hemorrhage was cleared. The fibrous membrane was removed and laser photocoagulation was performed. Mild vitreous cloudy was observed on the second day after surgery. No active hemorrhage was found through the scleral incision wounds. The visual acuity was 0.1 at 3 months.

## Discussion and conclusions

In general, longer sclera tunnels are associated with better wound closure, and less post-operative leakage. We made sclera incisions parallel to limbus using a biplanar technique, with sclera tunnels estimated at about 1.5–2 mm long.

Apart from the stepped incision technique, there is another difference between 20-gauge (20G) and small gauge vitrectomy: the conjunctiva is usually not opened in microincision vitrectomy surgery. Tiny bleeding from the cut edge of sclera incisions is common, both in 20G and small gauge vitrectomy, but arterial hemorrhage has never been observed in thousands cases of 20G vitrectomy performed by the same surgeon. We thought the bleeding was not likely from conjunctival or episcleral vessels because 1) the intraocular hemorrhage was observed first; 2) sub-conjunctival bleeding was not found upon incision, but observed soon after the incision cannular was removed; 3) bleeding from conjunctiva or episclera might not be pulsating; 4) bleeding from conjunctiva or episclera could be stopped spontaneously or by simple compression; 5) the bleeding volume from conjunctiva or episclera would be much smaller.

We deduced that the tunnel incision caused active hemorrhage was from long posterior ciliary artery or anterior ciliary artery, because 1) the superonasal incision seemed crossing 9 o’clock in case 1, and 3 o’clock in case 2, the insertion needle would accidentally injury ciliary artery; 2) the fact that it occurs in the superonasal rather than in the superotemporal quadrant seems like an extra argument. 3) the bleeding could not stop spontaneously, it supports the opinion that the hemorrhage at incision position was from injured artery; 4) moving the cannula by cutter during surgery was associated with more severe bleeding.

The posterior long ciliary artery or anterior ciliary artery might be the source of bleeding in our cases. The posterior long ciliary arteries pass through the sclera nasal and termporal to the optic nerve and run horizontally at first in scleral tunnel, and then in suprachoroidal space, as far forward as the posterior part of ciliary body. The anterior ciliary arteries pierce the sclera just in front of the insertions of the rectus muscles in a slightly oblique direction from posterior to anterior. Each rectus muscle has two anterior ciliary arteries, except the lateral rectus muscle, which has only one [7].

Placing cannula crossing or near the 3/9 o clock might happen in such situations: inserting cannula close to horizontal axis with a long scleral tunnel; the eyeball being rotated unintentionally before inserting the superior cannulas. It must be strongly stressed that inserting the needle at 3/9 o’clock should be prohibited during vitrectomy to avoid ciliary artery injury.

The active bleeding in our cases gained rapid control by relocating the involved cannula, elevating intraocular pressure and compressing the incision wound. The realization and proper treatment of such complication should be kept in mind in small gauge vitrectomy.

## Additional file

Additional file 1: Arterial bleeding related to small gauge incision, Video illustration of arterial hemorrhage caused by 23-gauge tunnel incision in a case with macular hole. (MP4 16533 kb)

## Abbreviations

20G: 20-gauge
23G: 23-gauge
BCVA: Best corrected visual acuity
